# Beta‐cell activity and development of Type 1 diabetes

**DOI:** 10.1111/apm.13441

**Published:** 2024-05-26

**Authors:** Rikke Thea, Karsten Buschard

**Affiliations:** ^1^ Bartholin Institute Rigshospitalet Copenhagen Denmark

**Keywords:** Beta cells, activity, Type 1 diabetes, antigen expression, prevention

## Abstract

Type 1 diabetes (T1D) is an autoimmune disease, resulting in diminished islet integrity and destruction of the insulin‐secreting beta cells. In this review, we investigate the intrinsic relationship between the development of T1D and the activity of the beta cells. The idea was initially hypothesized in 1982 that an increased beta‐cell activity would enhance the surface antigen expression and thereby attract the immune system. Later, other findings support this idea, including increased risk of T1D development during third trimester of pregnancy, and the difference in T1D incidence in Russian and Finnish Karelia due to different lifestyles. Other implications of high beta‐cell activity, such as reduced sulfatide levels, formation of non‐correct insulin molecules and an increase in IFN‐alpha upon virus attack, can contribute to the development of T1D. A possible way to prevent the development of T1D is to diminish beta‐cell activity, which has shown promising results in animal models.

## HISTORY

In 1982, when I (KB) wrote my doctoral thesis, I got the idea that high activity of the beta cells could be involved in development of Type 1 diabetes (T1D). An implication of this could be that the active beta cells exposed more antigens at their surface and thereby attract the immune system, which just at that time were realised to be part of their destruction. My mentor was Jørgen Rygaard, and I have been first author in 10 of his 44 papers in APMIS. Jørgen Rygaard liked good ideas, and we started to make a study using beta cells in high and low glucose concentration and stained them with an antibody specific for beta cells [1]. The result was obvious – the more active the greater the staining [2]. In another study, islets from Lewis rats were treated with different concentrations of glucose, and the result showed an increased antigen expression with increased glucose stimulation, thereby higher beta‐cell activity [3].

## BETA‐CELL ACTIVITY AND ANTIGENICITY

Our original study has later been confirmed, and an important utilization was that detection of islets cells antibodies is performed better when using pancreas sections from individuals having high beta‐cell activity [4]. Another study also showed that a high glucose concentration on rat islets, increased the expression of 64 000‐M_r_ autoantigen, now called glutamic acid decarboxylase (GAD) antigen [5].

In another study, we described an interesting outcome of the increased signalling from active beta cells of their antigens to the immune system. It had been found epidemiologically that children of T1D fathers have a risk of T1D at 6% whereas it is only 2–3% if a child has a T1D mother [6]. When the mother has diabetes, the blood sugar level is high also in the foetus and the foetal beta cells are matured early and produce a lot of insulin which normally is not the case. Since insulin is a trophic factor, the foetus grows faster and might be difficult to deliver late in pregnancy for which reason the diabetic mothers were giving birth in 37 week or by caesarean section. In these foetuses, the beta cells are more matured and express more adult antigens than seen for normal babies. We tried to mimic this situation by stimulating the beta cells neonatally in diabetes prone BioBreeding (BB) rats by injecting beta‐cell stimulatory agents like arginine, and the diabetes incidence among these rats was much lower than among controls [7]. We later did the same in non‐obese diabetic (NOD) mice for which similar result was seen although not as dramatic as for the BB rats [8]. The finding was interpreted that signalling more adult antigens neonatally would perform a better self‐recognition and thus a more difficult breakthrough for autoimmunity. Interestingly, we could show the same result for thyroiditis immunity [9].

## BETA‐CELL ACTIVITY AND PATHOGENIC IMPLICATIONS

The higher beta‐cell activity has some possible pathogenetic outcomes. In a study by Palmer et al., rat islets were exposed to IL‐1beta [10]. Simultaneously, the beta‐cell activity was modulated by either glucose or non‐glucose agents. The result showed an increased islet disintegration with increased IL‐1beta concentrations and beta‐cell activity. Thus, active beta cells are more sensitive to IL‐1beta mediated degradation than the resting beta cell: ‘the moving target’ [10].

Sulfatide is an important sphingolipid in the beta cells. Individuals developing diabetes have reduced sulfatide in the islets compared to healthy individuals [11], which possibly associates with the beta‐cell activity [12]. Sulfatide functions as a chaperone for insulin and is important for insulin folding and secretion. We examined the amount of sulfatide in active beta cells compared to inactive beta cells and found that there was a lower amount of sulfatide in the active beta cells [13]. Moreover, it was also found that sulfatide could inhibit specific T‐cell proliferations, suggesting that the inactive beta cells have an advantage against the immune system [13].

The T‐cell‐dependent destruction of beta cells is caused by recognition of specific molecules. Delong identified epitopes formed by cross‐linking proinsulin peptides to other peptides which is present in the secretory granules of the beta cells. These hybrid insulin peptides (HIPs) are antigenic for the T cells [14]. HIPs contain an insulin fragment which is linked to another secretory granule peptide, and by mass spectrometry, they have been detected in both mouse and human pancreatic islets [15].

Other molecules which give rise to T‐cell‐dependent destruction of the islets are defective ribosomal products (DRiPs). IFN‐gamma and not IFN‐alpha increases the expression of PSMB10, an important subunit of the immunoproteasome in endocrine beta cells. This upregulation of PSMB10 facilitates the presentation of insulin DRiP‐derived peptides to CD8^+^ T cells, potentially contributing to the autoimmune destruction of beta cells in T1D [16].

The reason for formation of HIPs and DRiPs remains unknown but it might be due to ER stress in the beta cell. ER stress can lead to beta‐cell destruction in the development of both T1D and T2D, but the term is used almost synonymously with beta‐cell stress [17].

During a virus attack on the cells, an increase in IFN‐alpha occurs. This elevated amount of IFN‐alpha activates 2′‐5′ oligoadenylate synthetase (OAS) which stimulates an increase in 2′‐5′‐linked oligoadenylate (2′‐5′ A). 2′‐5′ A activates RNAseL which cleaves ssRNA, preferably the virus' ssRNA, but a high activity of RNAseL will result in cleaving of the host's own ssRNA, then possibly destroying the beta cells [18]. Interestingly, there is a higher 2′‐5′ synthetase activity in beta cells compared to alpha cells [19]. OAS is regulated by phosphodiesterase 12 (PDE12) which degrades OAS, resulting in a decrease in RNAseL and thereby less cleaving of ssRNA. Interestingly, in a study we found a decrease in PDE12 in both newly diagnosed T1D patients and recently diagnosed T1D patients [20]. This could be explained by a poor regulation of OAS in T1D patients and thus an increase in RNAseL‐mediated destruction of the beta cells.

## BETA‐CELL ACTIVITY AND TYPE 1 DIABETES PATHOGENESIS AND PREVENTION

During pregnancy, there is an increased need for insulin, and we found a 3.8‐time increased incidence of true T1D in third trimester [21], which may be explained by the extra stress on beta cells in this period.

The prevalence of T1D is six times higher in Finnish Karelia compared to Russian Karelia. This could be due to the difference in lifestyle between these two countries despite being geographical next to each other. Finland is much more westernized with high consumption of refined carbohydrates and thereby more stress on the beta cells, which may contribute to the high incidence of T1D compared to Russia [22]. In addition, during second world war with low food supply, the diabetes incidence decreased in Germany possibly due to less activity of the beta cells [23].

A possible prevention of T1D is treatment with prophylactic insulin. In BB rats, the T1D incidence was reduced when treated with prophylactic insulin. Only 6 out of 36 BB rats were insulin‐dependent after treatment, compared to 15 out of 36 control rats [24]. Prophylactic insulin treatment in NOD mice showed a corresponding reduction in diabetes incidence [25], seen also in an adoptive cell transfer of diabetes [26]. Furthermore, a diabetic virus was less aggressive when the beta cells displayed low activity [27]. A human clinical trial with prophylactic insulin treatment showed no decrease in the incidence of T1D [28]. This might be due to the low dosage of insulin, which may not have been enough even to mice either. The insulin dose given was only 0.1 U/kg body weight whereas the dose given to BB rats in the original study was 15 U/kg body weight. A study later confirmed this theory by showing the low human dose did not have an effect in either NOD mice or BB rats [29]. In another clinical study, newly diagnosed insulin‐dependent diabetes mellitus patients received a higher dosage of insulin for 2 weeks than conventional treatment, to keep their blood glucose levels between 3.3 and 4.4. This treatment resulted in a higher plasma level of C‐peptide and better metabolic control a year after intervention, due to an improved beta‐cell function [30].

Toll‐like receptor 5 (TLR‐5) is found on the membrane of beta cells as part of the innate immune system and is activated during infections by flagellin. However, TLR‐5 is also activated upon glucose stimulation [31]. In mice, TLR‐5 deficiency showed to increase the development of T1D [32], and thus, induction of TLR‐5 seems to be protective.

Another way to prevent T1D could be fasting. In a study, we showed a reduction of T1D incidence in BB rats from 79% to 50% when food was withdrawn from the rats for 24 h every other day [33]. This reduces the stress on beta cells, and hence, short‐term fasting could prevent T1D.

## CONCLUSION

This review investigates the role of an increased beta‐cell activity in the development of T1D. It is shown that an increased beta‐cell activity can enhance the surface antigen expression on the beta cells, contributing to recognition of the immune system and thereby a T‐cell‐dependent destruction of the beta cells. The findings also suggest potential preventative strategies, including prophylactic insulin treatment in relevant doses, modulation of Toll‐like receptor 5 activity and fasting.

## CONFLICT OF INTEREST

The authors have declared that no conflict of interest exists.

## Data Availability

The data that support the findings of this study are available from the corresponding author upon reasonable request.
